# A Systematic Review of Severe Maternal Morbidity in High-Income Countries

**DOI:** 10.7759/cureus.29901

**Published:** 2022-10-04

**Authors:** Oleksandra Kaskun, Richard Greene

**Affiliations:** 1 Department of Obstetrics and Gynaecology, Cork University Hospital, Cork, IRL

**Keywords:** systematic review, pregnancy-related complications, high-income countries, near miss, maternal near miss, maternal morbidity, severe maternal morbidity

## Abstract

With declining maternal mortality rates in high-income countries (HICs), severe maternal morbidity (SMM) is becoming an important quality measure of maternal care. However, there is no international consensus on the definition and types of SMM. This study aims to critically analyze published literature on SMM in HICs. The objectives are to compare definitions and criteria used to identify SMM and identify the main causes and risk factors contributing to SMM in HICs. PubMed, Cumulative Index to Nursing and Allied Health Literature (CINAHL), and Scopus databases were searched for articles published between 2010 and 2022, results were filtered, and 10 studies were critically appraised. Six of the articles discussed SMM identification criteria and proposed definition modifications. Longer hospital stays and admission to the intensive care unit (ICU) were suggested as additional criteria. Disease-based criteria were shown to be superior to organ dysfunction criteria. Seven articles detailed common types of SMM as severe hemorrhage, hypertensive disorders, and preeclampsia/eclampsia. Six articles described SMM risk factors, of which advanced maternal age and cesarean delivery were the most common. This literature review identified disease-based criteria and Canadian study criteria as promising measures of SMM. It also identified several causes and risk factors of SMM common between HICs. These findings can help physicians identify women at risk of SMM. The study is however limited to eight HICs and 10 studies. Further research should aim to investigate how these criteria compare with previous sources of criteria and discern the association of weight and race risk factors with SMM.

## Introduction and background

Maternal health is an important measure of a country’s socioeconomic progress [1]. As maternal mortality rates have declined precipitously in high-income countries (HICs) to the level of becoming rare events, the World Health Organization (WHO) has suggested tracking the incidence of severe maternal morbidity (SMM) as a quality indicator of obstetric care [2]. The Maternal Morbidity Working Group organized by the WHO defines maternal morbidity as any chronic or acute health condition due to or aggravated by pregnancy or childbirth that has a negative impact on the woman’s well-being [1].

In contrast, there is no standardized definition of SMM or internationally consistent case identification criteria. SMM is usually described as a “maternal near miss” case, the near death of a woman who survived a complication relating to pregnancy or childbirth or within 42 days of termination of pregnancy [3,4]. The WHO proposed guidelines in 2011 for identifying maternal near miss cases based on clinical criteria, laboratory markers, and management proxies [4]. They included five potentially life-threatening conditions (severe postpartum hemorrhage, severe preeclampsia, eclampsia, sepsis, and ruptured uterus), a range of critical interventions or admission to the intensive care unit (ICU), and seven types of organ dysfunction as near miss criteria [4].

However, varying definitions of SMM and variations of case inclusion criteria have been used by hospitals and countries around the world. These variations can be the inclusion or exclusion of prepregnancy conditions or suggested expansions to either the 2011 WHO list or other country-specific lists of criteria [1]. The nonuniformity of the definition and the lack of consensus on inclusion criteria hamper comparative analysis and the determination of the true global burden of SMM.

The rates of SMM have not seen similar declines as have maternal mortality rates, and in some HICs such as the United States (USA), they have increased. According to the Centers for Disease Control and Prevention (CDC), the annual prevalence of SMM in the USA has more than doubled between 1998 and 2014 [5]. The apparent increase in SMM can be attributed to the changing characteristics of women giving birth over the last few decades: advanced maternal age, obesity, comorbidities such as diabetes or hypertension, and the increased occurrence of cesarean delivery. These factors have been associated with higher SMM risk [5], but the increase can also be due to changes in SMM identification criteria.

Aim and objectives

The aim of this study was to systematically analyze and critically appraise published literature on SMM in obstetrics in HICs. The specific objectives were to compare the definitions and criteria used to identify SMM in HICs, identify the main types of SMM in different countries, and identify the principal risk factors contributing to SMM.

## Review

Methods

Search Strategy

An electronic search was performed on July 28, 2022, using three databases, namely, PubMed, Cumulative Index to Nursing and Allied Health Literature (CINAHL), and Scopus, to identify relevant literature to answer the objectives of this review. The keywords used in different combinations were as follows: severe maternal morbidity, near miss, maternal near miss, developed countries, and high-income countries. The results were then filtered for publication between 2010 and 2022, free full text availability, availability in English, and academic journal type.

Study Selection

The initial PubMed search yielded 57 results, condensed to 27 after filters. CINAHL produced 169 results, 121 after filters. Scopus produced 745 results, 267 after filters. To supplement the search, three articles were added from the reference list of the other articles [5-7]. This resulted in 418 papers. Database results were combined using the reference manager Mendeley, yielding 365 papers after duplicate removal. Subsequent results were screened for eligibility by title and abstract according to the inclusion and exclusion criteria in Table 1. Papers published prior to 2010 were excluded as they would be less recent and guidelines for identifying SMM were updated in 2010. Study populations were limited to HICs, and the country’s income grouping was used as a marker of adequate healthcare in the country. Since this study focused on the criteria used to identify SMM, pregnancy type was limited to singleton to allow for consistent comparison between studies. Furthermore, studies that focused on maternal mortality or neonatal outcomes were excluded as the interest of this study is maternal morbidity.

**Table 1:** Summary of inclusion and exclusion criteria. HIC: high-income countries; SMM: severe maternal morbidity

Category	Inclusion criteria	Exclusion criteria
Publication date	2010-2022 (July 28)	Prior to 2010
Text availability	Free full text available	Unavailable free full text
Language	English	Not in English
Article type	Original research in academic journals	Systematic review, poster/conference, protocol, commentary article, literature review, case study, narrative review
Research location	2022 HICs as defined by World Bank	Not in HICs
Type of pregnancy	Singleton	Twin/multiple
Outcomes	Focused on SMM outcomes or factors	Out of scope or not focused on SMM outcomes, focused on maternal mortality outcomes, and focused on neonatal outcomes

The breakdown for reasons 330 papers were excluded from the review is presented in Table 2. Protocols, posters, reviews, case studies, and commentary articles were excluded with a preference for original research.

**Table 2:** Reasons for exclusion of articles after screening by title and abstract. SMM: severe maternal morbidity; HIC: high-income country

Reasons for exclusion	Number of articles
Studies out of scope/not focused on SMM outcomes	141
Not in HICs	87
Studies focused on maternal mortality outcomes	28
Studies focused on neonatal outcomes	16
Studies focused on twin/multiple births	4
Protocol	13
Poster/conference	11
Systematic review	16
Literature review	7
Commentary article	4
Case study	2
Narrative review	1
Total excluded	330

The remaining 35 articles underwent a full-text review, of which 25 were excluded for the reasons in Table 3. These exclusions were used as the goal was to capture studies that assess SMM as a concept and not individual events or conditions in the maternal population. Studies that only focused on a single SMM and single socioeconomic/ethnic factor or defining a single SMM criterion were excluded for this reason. A total of 10 articles were included in the review.

**Table 3:** Reasons for exclusion of articles after full-text review. SMM: severe maternal morbidity

Reasons for exclusion	Number of articles
Focus on the association of only one factor with SMM	13
Focus on the association of socioeconomic factors with SMM	5
Focus on the association of ethnic factors with SMM	2
Outcomes not reported in terms of SMM (substandard care or future progress focus)	2
Focus on model validation as outcomes	2
Focus on definitions of SMM criteria instead of which criteria to be included	1
Total excluded	25

**Figure 1:**
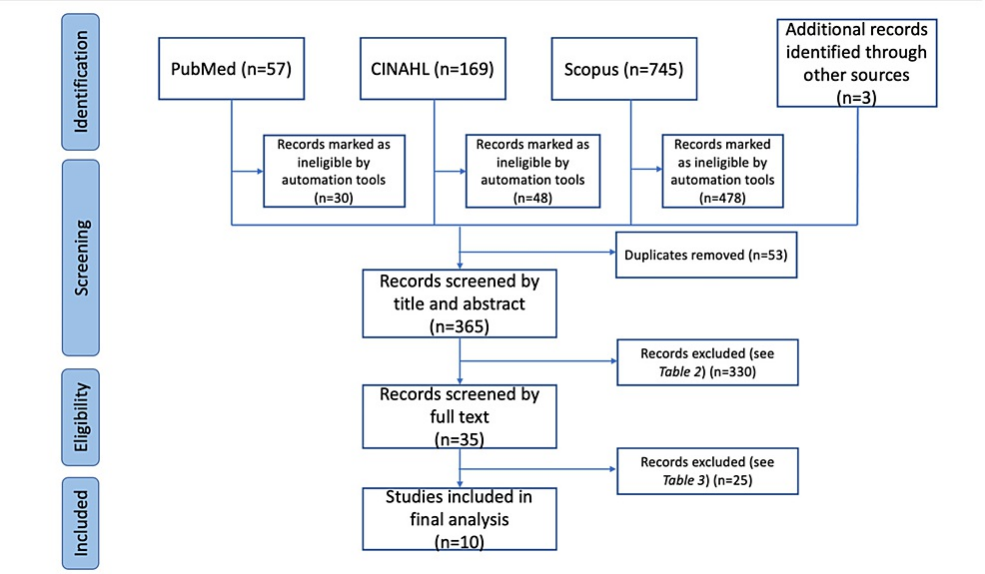
PRISMA flowchart. PRISMA: Preferred Reporting Items for Systematic Reviews and Meta-Analyses

Figure 1 illustrates the Preferred Reporting Items for Systematic Reviews and Meta-Analyses (PRISMA) diagram describing the selection process of articles [8].

Article Validity

The 10 articles selected for this review were critically evaluated using the Evidence-Based Librarianship (EBL) Critical Appraisal Checklist [9]. This tool evaluates the quality of a study, including the study population, method of data collection, study design, and results obtained.

Results

Summary Tables

From a total of 10 studies, there were six retrospective cohort studies [5,6,10-13], three prospective cohort studies [7,14,15], and one that had a combined type of first-half retrospective and second-half prospective cohort [16]. The studies were conducted across eight countries, the majority in the USA with three cases and one each in Canada [10], Australia [14], Ireland [7], Italy [15], Netherlands [13], and United Arab Emirates [16] (this is a HIC according to World Bank), and one study [11] covered three countries, namely, the USA, Australia, and England. The sample sizes ranged from 19 cases of SMM (among 2,773 live births) [14] to 47,973 cases of SMM (among 3,556,206 deliveries) [5]. Study periods ranged from six months [7,14] to 10 years [15]. A summary of the characteristics of the included studies is presented in Table 4.

**Table 4:** Summary of the characteristics of studies included in the systematic review (in alphabetical order). ICD-10: International Classification of Diseases and Health Related Problems, 10th Revision; CCI: Canadian Classification of Health Interventions; WHO: World Health Organization; ICD-9-CM: International Classification of Diseases and Health Related Problems, Ninth Revision, Clinical Modification; ICU: intensive care unit; HDU: high dependency unit; SMM: severe maternal morbidity

Author and year	Study type	Study dates	Study population	Sample size	Number of cases	Diagnostic criteria
Dzakpasu et al. (2020) [10]	Retrospective cohort	2012-2016	All hospital deliveries in Canada (excluding Quebec)	1,418,545	22,799	ICD-10 and CCI
Ghazal-Aswad et al. (2013) [16]	Retrospective cohort first three years and prospective cohort last three years	1998-2003	All births occurring in maternal units with over 500 births/year (four units) in the Emirate of Abu Dhabi	122,705	926	2011 WHO clinical criteria (eight conditions)
Jayaratnam et al. (2018) [14]	Prospective observational	December 1, 2014-May 31, 2015	All women admitted to King Edward Memorial Hospital during pregnancy or within 42 days of its termination	2,773	19	Clinical and biochemical parameter form based on the 2011 WHO criteria
Lazariu et al. (2017) [6]	Retrospective population-based observational study	2008-2013	All New York State female residents, 10-55 years old, who had live births at New York acute care hospitals	1,352,600	34,478	ICD-9-CM plus long hospital stay (at or above the 90th percentile) and admission to ICU
Leonard et al. (2019) [5]	Retrospective population-based cohort study	2007-2014	Live births in California with gestation >20 weeks	3,556,206	47,973	ICD-9-CM
Lipkind et al. (2019) [11]	Retrospective cohort	2008-2013	Delivery hospitalizations in large university hospitals in the USA, Australia, and England	516,781	4,333	ICD-10 in England and Australia, and ICD-9-CM in the USA
Mhyre et al. (2011) [12]	Retrospective cohort	2003-2006	Maternal hospital admissions for delivery in the Nationwide Inpatient Sample	3,463,327	4,550	ICD-9-CM
O’Malley et al. (2016) [7]	Prospective cohort	May 5-November 5, 2014	All women admitted to the Coombe Women and Infants University Hospital HDU	4,502	128	Scottish Audit and 2011 WHO criteria
Witteveen et al. (2016) [13]	Retrospective cohort	August 1, 2004-August 1, 2006	Women with SMM in the Netherlands	371,623	2,552	2011 WHO criteria and organ dysfunction criteria
Zanconato et al. (2019) [15]	Observational prospective study	2007-2016	Women admitted to the University Hospital of Verona	17,560	151	2011 WHO criteria (specifically the intervention-based and organ dysfunction criteria)

Critical Appraisal

The validity and quality of each study were reviewed in an objective and standardized manner, and the validity scores are presented in Table 5. The EBL Critical Appraisal Tool was used to evaluate these studies and can be found in the Appendix [9]. Each validity section contained several questions in the form of a checklist (Appendix) and required a “yes/no” answer. The “yes” responses were portrayed as a percent out of the total number of questions in the checklist category. All articles have section and overall scores above 75% and thereby were deemed valid.

**Table 5:** Validity scores calculated using EBL Critical Appraisal Tool. EBL: Evidence-Based Librarianship

Study	Population validity	Data collection validity	Study design validity	Results validity	Overall validity
Dzakpasu et al. (2020) [10]	100%	100%	100%	100%	100%
Ghazal-Aswad et al. (2013) [16]	100%	100%	100%	83%	96%
Jayaratnam et al. (2018) [14]	80%	100%	100%	83%	91%
Lazariu et al. (2017) [6]	100%	100%	100%	100%	100%
Leonard et al. (2019) [5]	100%	100%	100%	100%	100%
Lipkind et al. (2019) [11]	100%	100%	100%	83%	96%
Mhyre et al. (2011) [12]	100%	100%	100%	100%	100%
O’Malley et al. (2016) [7]	80%	100%	100%	83%	91%
Witteveen et al. (2016) [13]	100%	100%	100%	100%	100%
Zanconato et al. (2019) [15]	100%	100%	100%	83%	96%

A summary of the results, strengths, and limitations of the articles included in the study is detailed in Table 6. The results are then described under the headings of objectives 1, 2, and 3.

**Table 6:** Summary of the results, strengths, and limitations of the studies included in the systematic review. SMM: severe maternal morbidity; HELLP: hemolysis, elevated liver enzymes, low platelet count; ICU: intensive care unit; ICD: International Classification of Diseases and Health Related Problems; DIC: disseminated intravascular coagulation; HDU: high dependency unit; WHO: World Health Organization; SLE: systemic lupus erythematosus

Author and year	Results	Study strengths	Study limitations
Dzakpasu et al. (2020) [10]	1. The SMM rate was 16.1/1,000 deliveries. 2. The main types of SMM were severe preeclampsia and HELLP syndrome, severe postpartum hemorrhage, maternal ICU admission, and hysterectomy. 3. The SMM rate was higher in older women (>40 years old) and those with previous or current cesarean delivery. 4. Twelve SMM types were identified and 46 subtypes.	Very large sample size, multidisciplinary input, proposed new criteria for SMM, and long study period	Inability to identify some clinically relevant cases (severe obesity) and distinguish between preexisting and acute complications, and exclusion of Quebec
Ghazal-Aswad et al. (2013) [16]	1. The SMM rate was 7.5/1,000 deliveries. 2. The most common types of SMM were hypertensive disorders and hemorrhage.	Long study period, prospective design, and clearly defined clinical criteria	Old dataset, only one province was included, and limited to large maternity units
Jayaratnam et al. (2018) [14]	1. The SMM rate was 7/1,000 deliveries. 2. The main types of SMM were postpartum hemorrhage, preeclampsia, and early pregnancy complications.	Cases were reviewed independently by two investigators, and prospective design	Small sample, only one hospital, not nationally representative, and short study period
Lazariu et al. (2017) [6]	1. Case incidence of 2.55% (25.5/1,000) using the expanded criteria (3% increase in cases compared to using the ICD only). 2. The risk factors for SMM were identified as age of <20 or >35, underweight, cesarean delivery, and non-white race.	Very large sample size (including 93% of live birth records for New York State), expanded the ICD definition of SMM, and long study period	Hospital discharge records, not all complete, and accuracy varies by hospital; prepregnancy comorbidities were not consistently recorded
Leonard et al. (2019) [5]	1. The SMM rate was 13.5/1,000 deliveries. 2. Prepregnancy comorbidities and cesarean delivery were associated with SMM (twofold higher), advanced age was associated to a lesser degree, but prepregnancy obesity was not associated.	Very large, diverse sample size, linkage between vital records and patient records allowed the study of prepregnancy risk factors, and long study period	Observational study, data may lead to misclassification (prepregnancy weight was self-reported), limited to California, and included a limited number of risk factors
Lipkind et al. (2019) [11]	1. The overall SMM rate was 8.2/1,000 deliveries: 15.6 in the USA, 8.2 in Australia, and 5.0 in England. 2. The most common types of SMM were DIC, acute renal failure, cardiac event ventilation, hysterectomy, and eclampsia. 3. The risk factors associated with SMM were advanced maternal age (>40 years old), hypertension, diabetes, and substance abuse.	Large, international sample size, use of academic medical centers for consistency, and long study period	Hospital discharge coding (vary between countries and hospitals); limited number of hospitals, and countries are not equally represented; and only academic centers are included
Mhyre et al. (2011) [12]	1. Defined SMM (in addition to ICD measures) as an end-organ injury with a length of stay greater than 99th percentile or discharge to a second medical facility. 2. The SMM rate was 1.3/1,000 deliveries. 3. The risk factors contributing to the majority of SMM were comorbidities (pulmonary hypertension, malignancy, and SLE) and complications (DIC, acute liver disease, and acute respiratory distress syndrome).	Large, diverse, national sample size, expanded on the ICD-9 definition of SMM, and access to preexisting comorbidity data	Specific ICD codes do not exist for many conditions (placenta accreta), cannot study rare conditions, and old dataset
O’Malley et al. (2016) [7]	1. Of the 128 admissions to HDU, 16 women fulfilled the SMM criteria defined by the Scottish Audit, while eight met the WHO criteria; 83 women had severe maternal complications. 2. The common reasons for admission to HDU were hemorrhage, hypertension, and sepsis.	Two different SMM criteria to identify cases (Scottish Audit and WHO), and recent dataset	Short study period, small sample number (16 + 8 cases meeting the criteria), and limited to HDU admission
Witteveen et al. (2016) [13]	1. About 9% of cases identified as SMM in the LEMMoN study were missed using the WHO criteria. 2. Organ dysfunction criteria failed to identify ~60% of SMM cases. Disease-based criteria detected ~90% of SMM cases. 3. The most common types of SMM were postpartum hemorrhage, DIC, and admission to ICU. 4. The risk factors associated with SMM were identified as higher maternal age (35+ years) and long hospital stay.	Two independent investigators, discrepancies were discussed with team, and large sample size	Incomplete or missing information in the database (bias limited by team discussion) and older dataset
Zanconato et al. (2019) [15]	1. The SMM incidence rate was 8.6/1,000 deliveries. 2. The most common types of SMM were severe obstetric hemorrhage and hypertensive disorders. 3. The factors also associated with SMM were preterm birth, cesarean section, and sub-Saharan African origin.	Long study period and prospective design	Single institution and only intervention-based and organ dysfunction criteria were used to identify cases

Objective 1: Comparing SMM Definitions and Criteria

Six articles discussed SMM definitions and case criteria [6,7,10,12,13,15]. To identify SMM cases, two articles from the United States used the International Classification of Diseases, Ninth Edition (ICD-9) [6,12], one Canadian article used both the ICD 10th edition (ICD-10) and the Canadian Classification of Health Interventions (CCI) [10], two European articles used the WHO maternal near miss criteria [13,15], and one Irish article [7] used the WHO criteria alongside the Scottish Audit criteria [17]. All of these articles either commented on the use of the criteria to identify cases or expanded on the definitions/criteria of these systems.

Lazariu et al. expanded on the ICD-9 criteria for SMM cases by including a long hospital stay (at or above the 90th percentile) and admission to the intensive care unit (ICU) as part of the definition [6]. This resulted in a 3% increase in SMM cases compared to using ICD only. Mhyre et al. suggested a similar expansion on the ICD-9 definition, by adding end-organ injury with a length of stay greater than the 99th percentile or discharge to a second medical facility [12]. Dzakpasu et al. investigated a list of morbidity types and subtypes, their incidence, and their association with case fatality and length of hospital stay [10]. They evaluated 13 SMM types that were not a part of the ICD-10 or CCI, of which six were suggested for inclusion.

O’Malley et al. reported double the cases identified as SMM using the Scottish Audit criteria [17], compared to WHO criteria [7]. Zanconato et al. [15] and Witteveen et al. [13] both investigated the 2011 WHO SMM criteria. The former focused on using only the intervention-based and organ dysfunction criteria [15]. Meanwhile, the latter group used all three WHO criteria categories separately and suggested that disease-based criteria identified the most cases, while organ dysfunction criteria missed about 60% of SMM cases [13].

Objective 2: Main Types of SMM

Seven articles detailed the main types of SMM in their respective countries [7,9,11,13-16]. All but one [11] of these articles reported a severe obstetric hemorrhage as the main type of SMM. The Italian [15], Irish [7], and United Arab Emirates [16] studies reported hemorrhage and hypertensive disorders as the most common types of SMM. Meanwhile, the Canadian [10] and Dutch [12] studies reported it to be ICU admission. Other common SMM types between some studies were preeclampsia and/or eclampsia [10,11,14], disseminated intravascular coagulation (DIC) [11,13], and hysterectomy [10,11].

Objective 3: Principle Risk Factors of SMM

Six articles described risk factors associated with SMM [5,6,10-13]. The two most common risk factors were advanced maternal age [5,6,10,11,13] and a cesarean delivery [5,6,10,15]. Advanced maternal age was either defined as above age 35 or 40 depending on the study. Leonard et al. reported that SMM was two times higher among women with a cesarean delivery than vaginal delivery [5]. The study did not find an association between SMM and prepregnancy obesity [5], while Lazariu et al. reported an association between SMM and being underweight instead [6]. Two studies identified maternal comorbidities as a risk factor, which included conditions such as pulmonary hypertension, chronic renal disease, and malignancy [5,12]. Leonard et al. also reported that SMM was two times higher among women with comorbidities [5]. Two studies suggested non-white origin to also be a risk factor [6,15].

Discussion

This study looked at 10 international articles to ascertain the criteria used to identify SMM and identify the main types of and risk factors contributing to SMM among eight HICs.

There is no international consensus on which criteria to use to identify SMM. As seen in the results, the ICD and WHO are common sources of identification criteria for countries, with two additional sources being the CCI and the Scottish Audit [17]. Two articles with long study periods and large samples suggested expanding the ICD criteria to include longer hospital stay and admission to ICU/secondary medical facilities to more comprehensibly identify SMM cases [6,12]. The Canadian study evaluated the ICD-10, CCI, and new measures to propose a master list of types and subtypes that can be used to identify SMM [10]. They suggested adding the following types of SMM to the ICD-10 list: severe preeclampsia, HELLP syndrome, acute fatty liver, red blood cell transfusion, ICU admission, and inversion of the uterus [10]. This study had a large, diverse sample allowing external validity, is recent (2019), demonstrates extensive research and clarity, and used a multidisciplinary team to limit bias. According to the WHO, organ dysfunction criteria are the most promising markers to detect SMM [13]. However, as demonstrated by Witteveen et al. [13] (and seen to an extent in O’Malley et al. [7]), these criteria missed 60% of cases, and instead, disease-based criteria warrant further attention. Thereby, these findings provide guidance on how to achieve a representative definition of SMM. Specifically, disease-based criteria and the measures identified by the Canadian study should be used as the basis for future identification of SMM.

As HICs use different criteria to identify SMM cases and consist of a differing composition of individuals, the main types and risk factors of SMM were compared between countries. Many similarities were nevertheless seen between the principal types and risk factors affecting women in the different HICs. This suggests a few common factors that need to be addressed and monitored to limit SMM in the future. The most common types of SMM were severe hemorrhage as identified by six articles [7,10,13-16], hypertensive disorders named by three articles [7,15,16], and preeclampsia/eclampsia also by three [10,12,14]. Of these articles, the article by O’Malley et al. is of a lower quality mainly due to the small sample size and limitation to the HDU, which reduces external validity and the ability to draw generalizable conclusions [7]. However, it remains in agreement with the other articles on the principal types of SMM.

The most common risk factors between countries were advanced maternal age [5,6,10,11,13] and cesarean delivery [5,6,10,15]. Two studies also suggested prepregnancy comorbidities such as pulmonary hypertension, malignancy, and systemic lupus erythematosus as risk factors [5,12]. An issue that arose was the differing definition of advanced maternal age, as it was either above 35 or above 40 years. It is important to establish a consensus on this factor for improved study comparability and clinical case screening. The association of the above risk factors with SMM was identified in previous literature [5,18], with the addition of obesity as a factor. However, Leonard et al. did not find this association [5], and being underweight was suggested as a factor instead [6]. The role of prepregnancy weight in SMM requires further study. Two studies also suggested race to be a risk factor, specifically non-white origin [6,15]. One study was from New York State [6], using a very large, diverse sample, and the other was Italian [15], using a sample of just over 100 SMM cases at a single institution (lacking external validity). However, racial disparity was also documented in two other studies, which reported increased SMM among non-western immigrant women [19] or sub-Saharan African women [20]. The role of race should be further investigated as a risk factor, and to determine if this is a consistent finding among various countries or if it is a bias due to confounding socioeconomic status.

Strengths and Limitations

The strength of this literature review is the inclusion of a variety of international articles representing eight HICs, most of which have large sample sizes and all of which were critically appraised to be over 90% valid. Furthermore, using three databases provided access to over 900 studies, and the systematic approach allowed reproducibility.

Limitations include being limited to only 10 articles and eight HIC, and inclusion was limited to free full texts in English, as additional studies were of interest but were not accessible. Additionally, two of the included studies, despite having a high validity, had small numbers of SMM cases [7,14].

Future Investigations

Disease-based criteria and the Canadian study criteria provide a more comprehensive insight into SMM. These measures should be further studied in other HICs to determine how they compare with previous sources of criteria. Additionally, future investigation into the association of risk factors with SMM, particularly weight and race, is required to improve early screening for SMM cases.

## Conclusions

SMM is an important measure of maternal quality of care, yet there is no international consensus on which criteria to use to identify SMM. This literature review sought to bridge this gap and was able to identify disease-based criteria and the Canadian study criteria as promising measures of SMM. Despite the differences in the criteria used between HICs, similar principal types of SMM were identified: severe hemorrhage, hypertensive disorders, and preeclampsia/eclampsia. Furthermore, common risk factors were also identified among the countries (advanced maternal age and cesarean delivery) that can assist with screening and identifying potential cases at risk of SMM. A consensus on defining SMM should be reached to allow obstetricians to identify patients at risk of SMM and practice improved preventative medicine.

## Appendices

The EBL Critical Appraisal Tool used to evaluate the article included in the present study is presented in Table 7.

**Table 7:** Evaluating the validity and quality of studies using the EBL Critical Appraisal Checklist. EBL: Evidence-Based Librarianship; Y: yes; N: no; U: unclear; N/A: not applicable; Y/total: %

EBL Critical Appraisal Checklist [17]		Dzakpasu et al. (2020) [10]	Ghazal-Aswad et al. (2013) [16]	Jayaratnam et al. (2018) [14]	Lazariu et al. (2017) [6]	Leonard et al. (2019) [5]	Lipkind et al. (2019) [11]	Mhyre et al. (2011) [12]	O’Malley et al. (2016) [7]	Witteveen et al. (2016) [13]	Zanconato et al. (2019) [15]
Section A: Population	Is the study population representative of all users who might be included in the study?	Y	Y	Y	Y	Y	Y	Y	Y	Y	Y
	Are inclusion and exclusion criteria clearly outlined?	Y	Y	Y	Y	Y	Y	Y	Y	Y	Y
	Is the sample size large enough to obtain precise estimates?	Y	Y	N	Y	Y	Y	Y	N	Y	Y
	Is the response rate sufficient for precise estimates?	N/A	N/A	N/A	N/A	N/A	N/A	N/A	N/A	N/A	N/A
	Is the choice of population free from bias?	Y	Y	Y	Y	Y	Y	Y	Y	Y	Y
	If a comparative study: Were participants randomized? At baseline, were the groups comparable? If not, was this addressed in the analysis?										
		N/A	N/A	N/A	N/A	N/A	N/A	N/A	N/A	N/A	N/A
		N/A	N/A	N/A	N/A	N/A	N/A	N/A	N/A	N/A	N/A
		N/A	N/A	N/A	N/A	N/A	N/A	N/A	N/A	N/A	N/A
	Was there informed consent?	N/A	Y	Y	N/A	N/A	N/A	N/A	Y	Y	Y
Section B: Data collection	Are data collection methods reported clearly?	Y	Y	Y	Y	Y	Y	Y	Y	Y	Y
	If a face-to-face survey, were interobserver and intraobserver bias reduced?	N/A	N/A	N/A	N/A	N/A	N/A	N/A	N/A	N/A	N/A
	Is the data collection instrument validated?	Y	Y	Y	Y	Y	Y	Y	Y	Y	Y
	If based on commonly obtained statistics, are they free from subjectivity?	N/A	N/A	N/A	N/A	N/A	N/A	N/A	N/A	N/A	N/A
	Is the outcome measured at an appropriate time for reporting the intervention’s effect?	Y	Y	Y	Y	Y	Y	Y	Y	Y	Y
	Is the instrument included in the publication?	Y	Y	Y	Y	Y	Y	Y	Y	Y	Y
	Are questions presented sufficiently clear in order to obtain accurate answers?	Y	Y	Y	Y	Y	Y	Y	Y	Y	Y
	Were those involved in data collection not involved in supplying a service to the target population?	Y	Y	Y	Y	Y	Y	Y	Y	Y	Y
Section C: Study design	Is the study type/methodology operated appropriate?	Y	Y	Y	Y	Y	Y	Y	Y	Y	Y
	Is there face validity?	Y	Y	Y	Y	Y	Y	Y	Y	Y	Y
	Is the research methodology precisely reported at a level that would permit its replication?	Y	Y	Y	Y	Y	Y	Y	Y	Y	Y
	Was ethical approval granted?	N/A	Y	Y	N/A	N/A	N/A	N/A	Y	Y	Y
	Are the outcomes clearly reported and discussed regarding the data collection?	Y	Y	Y	Y	Y	Y	Y	Y	Y	Y
Section D: Results	Are all the results clearly reported?	Y	Y	Y	Y	Y	Y	Y	Y	Y	Y
	Are confounding variables accounted for?	Y	Y	Y	Y	Y	Y	Y	Y	Y	Y
	Do the conclusions reflect the analysis accurately?	Y	Y	Y	Y	Y	Y	Y	Y	Y	Y
	Is subset analysis a minor, rather than a major, focus of the article?	Y	Y	Y	Y	Y	Y	Y	Y	Y	Y
	Are suggestions provided for further areas to research?	Y	Y	Y	Y	Y	Y	Y	Y	Y	Y
	Is the study externally valid?	Y	N	N	Y	Y	U	Y	N	Y	N
